# Effects of prebiotic galacto-oligosaccharide on postoperative cognitive dysfunction and neuroinflammation through targeting of the gut-brain axis

**DOI:** 10.1186/s12871-018-0642-1

**Published:** 2018-11-30

**Authors:** Xu-Dong Yang, Li-Kuan Wang, Hai-Yin Wu, Liang Jiao

**Affiliations:** 1 Department of Anesthesiology, Peking University School and Hospital of Stomatology, #22 Zhongguancun South Avenue, Beijing, 100081 China; 2 National Engineering Laboratory for Digital and Material Technology of Stomatology, Beijing Key Laboratory of Digital Stomatology, #22 Zhongguancun South Avenue, Beijing, 100081 China

**Keywords:** Postoperative cognitive dysfunction, Prebiotic, Neuroinflammtion, Gut-brain axis

## Abstract

**Background:**

Surgery-induced neuroinflammation plays an important role in postoperative cognitive dysfunction (POCD). Gut microbiota is a key regulator of neurological inflammation. Nurturing with prebiotics is an effective microbiota manipulation that can regulate host immunity and cognition. The aim of the present study was to test whether administration of the prebiotic Bimuno® (galactooligosaccharide (B-GOS) mixture) could ameliorate POCD and attenuate surgery-induced neuroinflammation through the microbiota-brain-axis.

**Methods:**

Adult rats undergoing abdominal surgery under isoflurane anesthesia were fed with water or prebiotic B-GOS supplementation (15 g/L) for 3 weeks. Novel objective recognition task was employed for testing cognitive changes on postoperative day three. Expression of microglial marker Iba-1 in the hippocampus was assessed by immunohistochemical staining. Expression levels of phenotypic gene markers of activated microglia (M1: iNOS, CD68, CD32; M2: Ym1, CD206, and SOCS3) in hippocampus were determined by quantitative polymerase chain reaction (qPCR). Inflammatory cytokines in the hippocampus were assessed using enzyme-linked immunosorbent assay (ELISA). Feces were collected for microbial community analysis.

**Results:**

Rats exhibited an impairment in novel objective recognition 3 days after surgery compared with control rats (*P* < .01). In the hippocampus, expressions of Iba-1 and M1 markers of surgical rats were significantly upregulated. Similarly, expressions of SOCS3 and CD206 in the hippocampus were upregulated. Additionally, increasing levels of IL-6 and IL-4 were evident in the hippocampus. Administration of B-GOS significantly alleviated cognitive decline induced by surgery (*P* < .01). B-GOS-fed rats showed a significantly downregulated activation of microglia and expressions of M1-related genes and SOCS3 and IL-6. While there was no significant difference in expressions of CD206 and Ym1 and IL-4 between the surgical and B-GOS groups. Analysis of gut microbiome found that administration of B-GOS induced a significant change beta diversity of the gut microbiome and proliferation of *Bifidobacterium* and other potentially anti-inflammatory microbes.

**Conclusions:**

Administration of B-GOS has a beneficial effect on regulating neuroinflammatory and cognitive impairment in a rat model of abdominal surgery and was associated with the manipulation of gut microbiota.

## Background

Postoperative cognitive dysfunction (POCD) is an adverse complication associated with anesthesia and surgery with an incidence of 11.7 to 60% [1]. It can occur in patients of all ages and following cardiac and non-cardiac surgeries, as well as after procedure sedation [2]. The mechanisms of POCD have not been elucidated fully. Preclinical and human experiments indicate that neuroinflammation plays an important part in the progression of POCD [3, 4].

Microglia are a specialized macrophage population in the central nervous system (CNS). These cells can be activated by insult factors and are the first cells to induce the neuroinflammatory response [5]. Studies have found that surgery is associated with microglial activation and inflammation-related cytokines in brain areas that are related to cognitive decline [6, 7]. In addition, microglia are highly plastic cells that can differentiate into complex phenotypes depending on specific microenvironmental signals within the brain. Two activated phenotypes are characterized: classically-activated (M1) with destructive pro-inflammatory and cytotoxic properties; alternatively-activated (M2) with the main function of repair, tissue remodeling (M2a), and anti-inflammation (M2b/c) [8, 9]. Therefore, microglia are a double-edged sword for neuroinflammation. Moreover, strategies based on phenotypic modulation of microglia have been found to attenuate postoperative cognitive decline in rodents [10, 11].

Mounting evidence from animal and human investigations indicate that the function and microenvironment of the brain are substantially influenced by gut microbes. The connection between intestinal microbes and the brain is known as the gut-brain axis [12]. Furthermore, gut microbiota is a key regulator of neuroinflammation and the activation of microglia are also regulated by gut microbiota [13, 14]. Therefore, regulation of gut microbiota may be a potential treatment for various neurologic diseases [15]. Prebiotics are a kind of substrate that can be selectively utilized by host microorganisms, leading to stimulation of gastrointestinal microbiota and conferring a health benefit [16]. There is increasing evidence that a diet with prebiotics has beneficial effects on host immunity and the gut-brain axis [17, 18].

Bimuno® (galacto-oligosaccharide (B-GOS) mixture) is a widely investigated specific nondigestible galacto-oligosaccharide especially potent in selectively promoting the proliferation of *Bifidobacterium* [19]. Previous studies found that B-GOS led to modulation of cortical inflammatory cytokine expression and increased the expression of brain-derived-neurotrophic factor through the manipulation of gut bacteria [20]. These effects were found to ameliorate the anxiety-like behavior induced by neuroinflammation [21]. However, the effects of prebiotic administration on POCD have not been extensively explored. The aim of the current research was to investigate whether administration of B-GOS could attenuate surgery-induced cognitive dysfunction in parallel with changes in inflammatory responses in the hippocampus, activation and phenotypic transformation of microglia, and the impact of gut microbiota composition.

## Methods

### Experimental animals and B-GOS supplementation

SPF Sprague-Dawley male rats (8 months old) were obtained from Beijing Vital River Experimental Animals Co. and housed under 12:12 light:dark cycle (20 ± 2 °C, humidity 50 ± 10%) conditions. Rats were fed standard chow and water ad libitum or a B-GOS (Bimuno®, Clasado Biosciences, UK) solution according to group allocations. B-GOS solution was prepared at a concentration of 15 g/L using sterile water. All experimental procedures were conducted in accordance with animal care guidelines and approved by the Ethics Committee of the Peking University Health Science Center (LA2016320).

### Experimental protocol

We first performed a pilot study to investigate the time course of postoperative cognitive impairment in the surgical model. Briefly, 12 rats were subjected to abdominal surgery under isoflurane inhaled anesthesia. Novel objective recognition test was assessed during postoperative days 3, 7, and 14. Four naïve control rats underwent behavioral assessments together with the 3 surgical groups. Results of the pilot study indicated the novel object preference ratio was significantly decreased 3 days after surgery (*P* < .01). However, the recognition ratio returned to normal on postoperative day 7 (Fig. 2A).

**Fig. 1:**
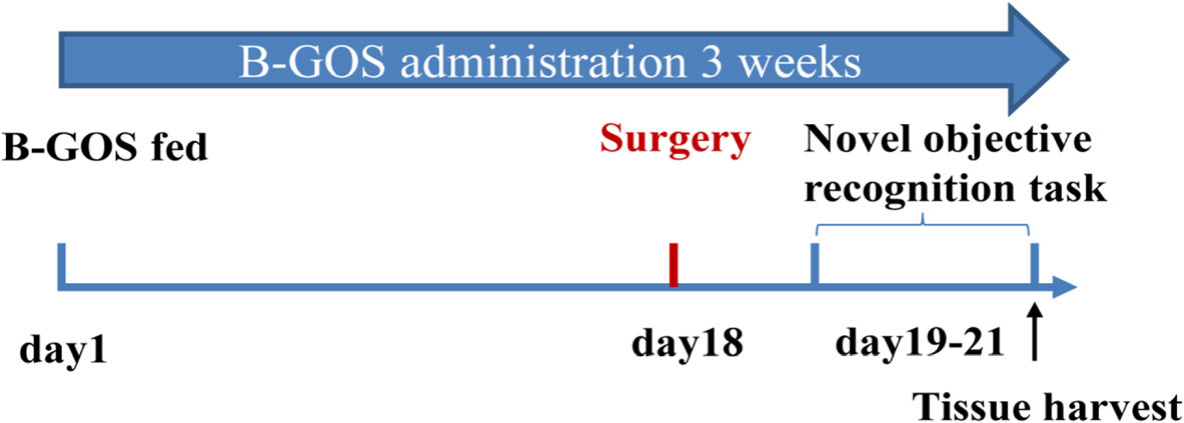
Experimental schedule of study. Thirty rats received standard chow with either normal drinking water (*n* = 20) or B-GOS solution (*n* = 10) for 21 days. On day 18, 10 water-fed rats and 10 B-GOS-fed rats received abdominal surgery under inhaled anesthesia. Novel objective recognition task was performed on days 19 to 21. Rats were sacrificed after completing the behavior test

The schematic of the study is presented in Fig. 1. Thirty rats were randomly allocated to normal drinking water and non-surgery group (Control group, C group), normal drinking water and surgery group (Surgery group, S group), or B-GOS solution and surgery group (Surgery + BGOS group, SG group), 10 rats per group. Rats in the S group and SG group received abdominal surgery under isoflurane inhaled anesthesia. Rats in the Control group did not receive anesthesia or surgery. Novel objective recognition of rats was determined on postoperative day 3. Rats in all groups were sacrificed after the behavior test. Brains were extracted and fixed in 4% paraformaldehyde in preparation for immunohistochemical staining. For quantitative polymerase chain reaction (qPCR) and ELISA, the hippocampi were collected and immediately frozen in liquid nitrogen, then stored at − 80 °C. Fecal samples were collected in individual sterile EP tubes and stored at − 80 °C.

### Surgery

Abdominal surgery with laparotomy combined with mesenteric ischemia-reperfusion was performed based on previous reports [22, 23]. These surgical procedures are known to result in postoperative cognitive dysfunction and neuroinflammation and are considered a model to mimic major abdominal surgery in humans [24, 25]. Briefly, rats were anesthetized under 2% isoflurane and oxygen. After the surgical area was sterilized, a vertical incision was made and the gastrointestinal tract was exteriorized and the upper mesenteric artery was reversible clamped for 30 min. During the clamping period, the small intestine, liver, colon, and stomach were gently manipulated by a sterile probe. The muscle wall and skin were closed by sterile suture. The abdominal incision was infiltrated with 2% lidocaine solution after suturing to treat skin incision pain. Body temperature was maintained at 37 ± 0.5 °C with a warm blanket. All operative procedures were performed by the same team.

### Novel object recognition test

Novel object recognition test is a useful tool to assess visual and spatial short-term memory for investigating the learning and memory of rodents [26]. In our experiment, rats were acclimated to the testing room and text box (60 cm × 60 cm × 40 cm) for 15 min on 1 day prior to exploration phase. Twenty-four hours later, rats were allowed to explore for 5 min two identical objects placed in the box (exploration phase). Rats that spent less than 30 s exploring both objects during the exploration phase were removed from further analysis. After 24 h, one of the objects was replaced by a different object. Rats were allowed to explore these objects for 5 min (novel object recognition phase). Time the rats spent exploring each object was recorded with two stopwatches by two investigators blind to the animals’ allocations. The novel object preference ratio was calculated as the time spent exploring the novel object, divided by the total time spent exploring the two objects.

### Immunohistochemical staining

Tissue sections of the brain containing the hippocampal CA1 area were cut from paraffin-embedded brain tissues and prepared for immunohistochemical staining using the Ready-to-Use Immunohistochemistry Hypersensitivity UltraSensitive™ S-P kit (Fuzhou Maixin Biotech, Fuzhou, China) according to the manufacturer’s instructions. After deparaffinization and rehydration in graded alcohols, antigen retrieval was performed with citrate buffer for 10 min at 90 °C. Next, gradually cooling at room temperature, sections were washed thrice with PBS followed by addition of 3% H_2_O_2_ for 10 min at room temperature to block endogenous peroxidase activity. The sections were then washed thrice with PBS and blocked with goat serum for 10 min at room temperature. After the goat serum was wiped, the sections were intubated with 1:800 rabbit-anti Iba-1(Fujifilm Wako Pure Chemical Corporation, Osaka, Japan) at 4 °C overnight, followed by intubation with biotin-labeled goat anti-rabbit secondary antibody for 10 min and streptomycin anti-biotin peroxidase for 10 min at room temperature. Next sections were labeled with 3,3′-diaminobenzidine, followed by hematoxylin counterstaining. Lastly, after washing, sections were dehydrated through gradients of ethanol and xylene. Number of Iba-1 positive cells was calculated per three random microscopic fields by two investigators blind to the allocation. Three brain slices per animal were chosen for analyses.

### Quantitative PCR analysis for microglia phenotypic genes

Total RNA was extracted from the whole hippocampus using Trizol reagent (Thermo Fisher Invitrogen Life Technologies, Waltham, MA, USA). Isolated RNA was reverse transcribed into cDNA with RevertAid First Strand cDNA Synthesis Kit (Thermo Fisher). Quantitative polymerase chain reaction (qPCR) was performed using designed primers (Table 1) and SYBR® Green Master (Roche Bioscience, Indianopolis, IN, USA) for 95 °C for 10 min and 40 cycles, then at 95 °C for 15 s and 60 °C for 60 s. Relative gene expression was calculated relative to the expression of glyceraldehyde-3-phosphate dehydrogenase (GAPDH) mRNA.

**Table 1:** Gene sequences of primers

Gene	Forward sequence	Reverse sequence
iNOS	*TTCACGACACCCTTCACCAC*	*AGCTGGTAGGTTCCTGTTGTTTC*
CD206	*TGTTTTGGCTGGGACTGACCTA*	*CGGGTGTAGGCTCGGGTAGTAG*
CD32	*CAAGAGCCCAAATCCAGCAGT*	*TTGAGATAGACCAAGGATACCAGG*
CD68	*CTGTATTGAACCCGAACAAAACC*	*GAGAATGTCCACTGTGCTGCTT*
SOCS3	*GCCACTTCTTCACACTGAGCGT*	*GAAGGTTCCGTCGGTGGTAAAG*
Ym1	*TCTGAATGATGGAGCCACTGATCT*	*GTCCTTGAGCCACTGAGCCTTA*
GAPDH	*TGGAGTCTACTGGCGTCTT*	*TGTCATATTTCTCGTGGTTCA*

### ELISA assays

The same weighted hippocampal tissues were homogenized in 4 °C normal saline (1,9 m/v) and centrifuged at 3500 rpm for 15 min to obtain sample supernatants. The levels of hippocampal IL-6 and IL-4 were detected using the Rat IL-6 ELISA-kit and Rat IL-4 ELISA-kit (MultiSciences, Hangzhou, China) according to the manufacturer’s instructions.

### Absolute quantification of *Bifidobacterium*

qPCR was performed to examine the fecal *Bifidobacterium* of rats. DNA was extracted from feces using a commercially-available stool DNA kit (TIANGEN, Beijing, China) according to the manufacturer’s instruction. The PCR reactions were performed in a volume of 20 μl containing 10 μl SYBR Green PCR Master Mix (Takara Bio USA, Mountain View, CA, USA), 1 μl of 10 μM primers, and 2 μl of cDNA. qPCR was performed using the following parameters: 95 °C for 30 s and 40 cycles with 95 °C for 5 s and 60 °C for 40 s. The primer used for the quantification of *Bifidobacterium* was F(5′-*GATTCTGGCTCAGGATGAACGC*-3′) and R(5′-*CTGATAGGACGCGACCCCAT*-3′). The gene copy numbers were quantified by comparing standard serial dilutions from 10^1^ to10^5^ copies of plasmid DNA. Results were expressed as copies of DNA per gram of stool and Log10 transformed for statistical analysis.

### High-throughput analysis of fecal microbial community

DNA from feces was extracted using a commercially-available stool DNA kit (Omega Bio-Tek, Norcross, GA, USA) following the manufacturer’s instructions. Purity and quality of the genomic DNA were checked on 0.8% agarose gels. Amplification of 16S rRNA gene was performed with the primers 357F(*ACTCCTACGGGAGGCAGCAG*) and 806R(*GGACTACHVGGGTWTCTAAT*), which target the V3-V4 hypervariable region. All samples were deep sequenced on a Miseq platform using the Illumina Analysis Pipeline version 2.6 (Illumina, San Diego, CA, USA). After screening the raw data by excluding low-quality reads, such as sequences shorter than 200 bps, low-quality scores (≤ 20), contained ambiguous bases, or did not exactly match primer sequences and barcode tags, the high-quality reads were separated using sample-specific barcode sequences and trimmed with Illumina Analysis Pipeline Version 2.6. Next, the dataset was analyzed using QIIME (http://qiime.org), the online bioinformatics pipeline that performs microbiome analysis from raw DNA sequencing data. The sequences at a similarity level of 97% were clustered into operational taxonomic units (OTUs), to generate rarefaction curves and to calculate the richness and diversity indices. All sequences were classified into different taxonomic groups using the Ribosomal Database Project Classifier tool. To evaluate α diversity of the gut microbiome, chao1 index was calculated. Principal coordinate analyses (PCoA) based on the unweighted UniFrac distances was performed in QIIME to examine the similarity between different samples and determined using PERMANOVA analysis.

### Statistics

Data were expressed as the mean and standard error. For multiple comparisons, a one-way ANOVA followed by posthoc Tukey test were performed. The Kruskal-Wallis and Dunn’s tests were used for nonparametric comparison. *P* < .05 was regarded statistically significant. Statistical analysis was performed with Graphpad Prism software (GraphPad Software, La Jolla, CA, USA).

## Results

### Cognitive changes after surgery

**Fig. 2:**
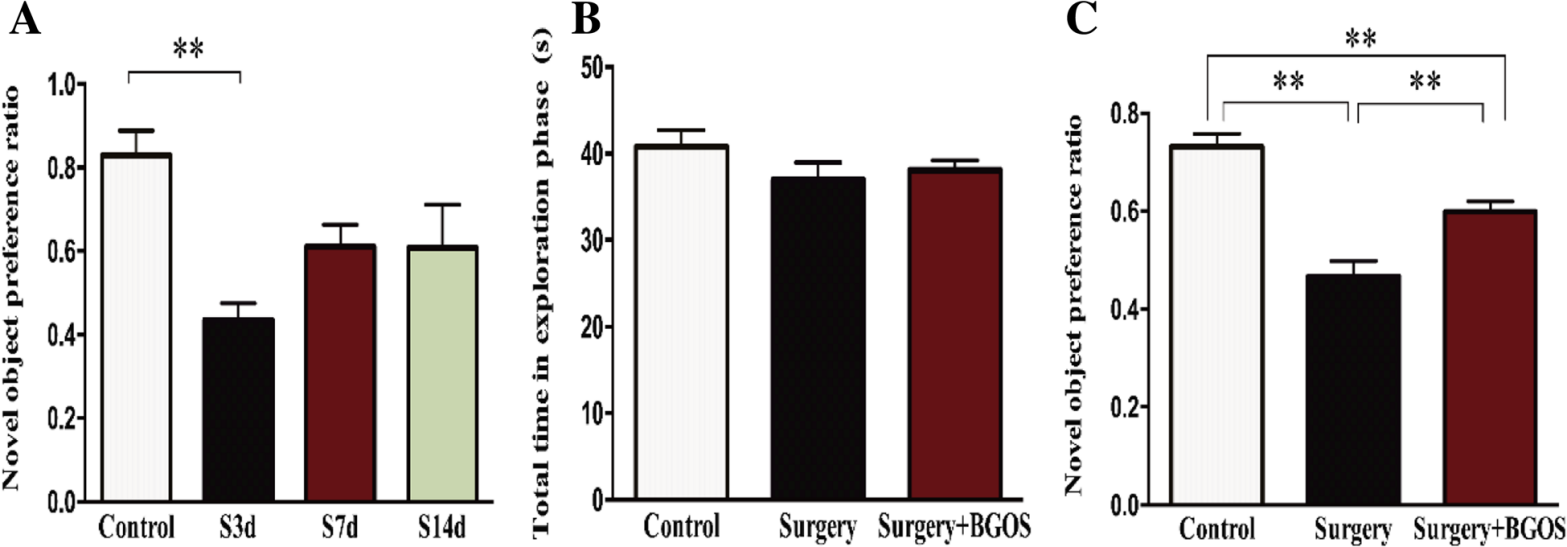
Time course of POCD and effects of B-GOS on surgery-induced impairment of novel objective recognition. Data are presented as mean ± SEM. (**a**) Time course of novel objective recognition following surgery. (**b**) Total exploration time of total objects during exploration phase. (**c**) Novel object preference ratio of recognition phase. S3d, S7d, and S14d = rats that underwent behavioral testing during days 3, 7, and 14 after surgery. **P < .01 for comparisons shown

Two rats (1 in S group and 1 in SG group) were excluded due to insufficient time spent on total objects during the exploration phase. The time spent on total objects in exploration phase did not differ significantly between groups (Fig. 2B). Novel object recognition index was significantly decreased 3 days after abdominal surgery (*P* < .01). Rats in SG group showed significantly improved cognition compared with Surgery group rats (*P* < .01) (Fig. 2C).

### Iba-1 expression in the CA1 area of the hippocampus

**Fig. 3:**
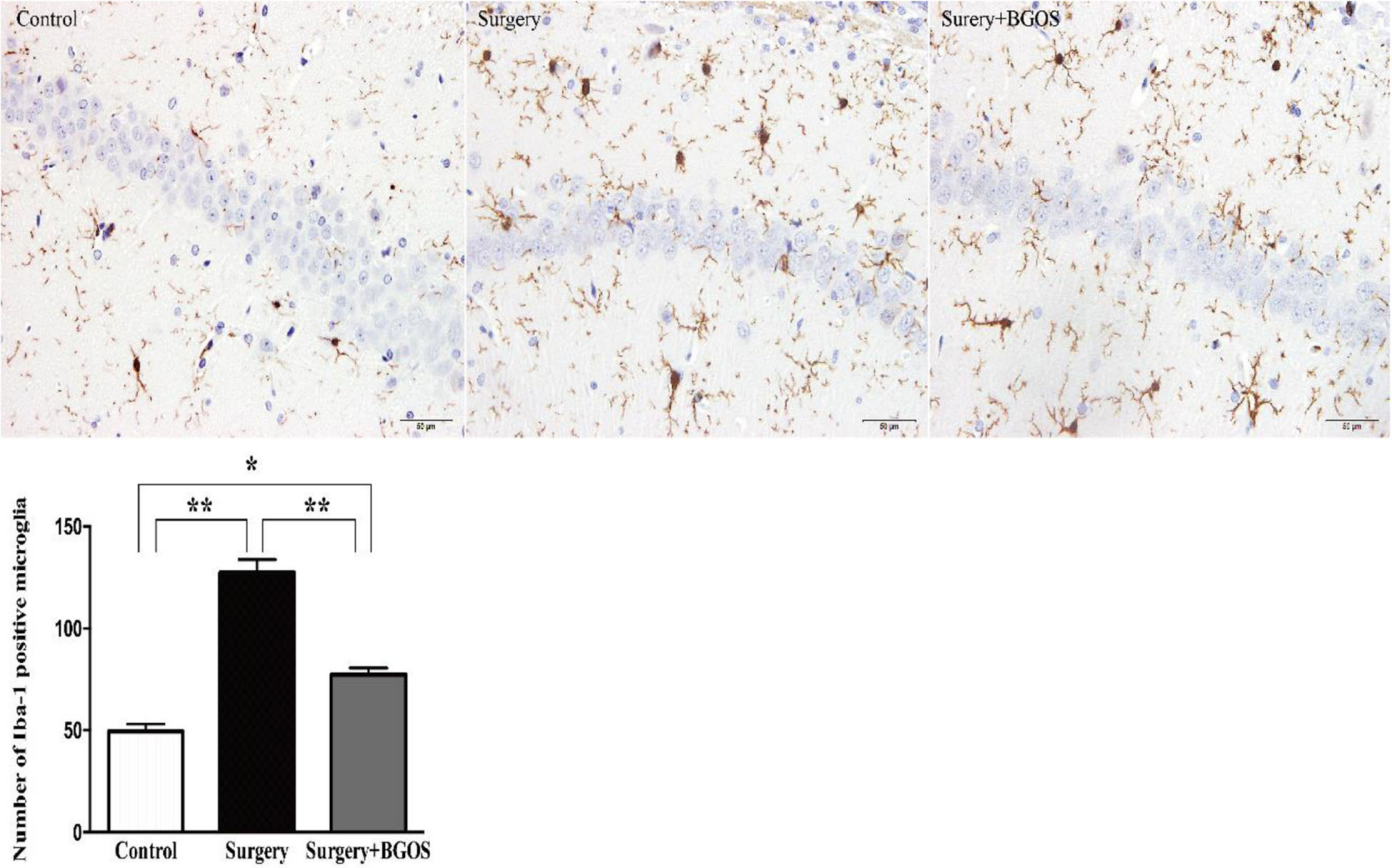
Effects of B-GOS on surgery-induced expression of Iba-1 in the area of hippocampal CA1. Data are presented as mean ± SEM (3 brain slices per rat, 3 rats per group). *P < .05 and **P < .01 for comparisons shown

Results from immunohistochemical staining indicated that the Iba-1 positive cell was significantly increased in the CA1 area of the hippocampi of rats in the Surgery group (*P* < .01). B-GOS-fed rats show a reduced Iba-1 positive cell in the hippocampal CA1 area after surgery (*P* < .01) (Fig. 3).

### Expressions of phenotypic-related genes of activated microglia in hippocampus

**Fig. 4:**
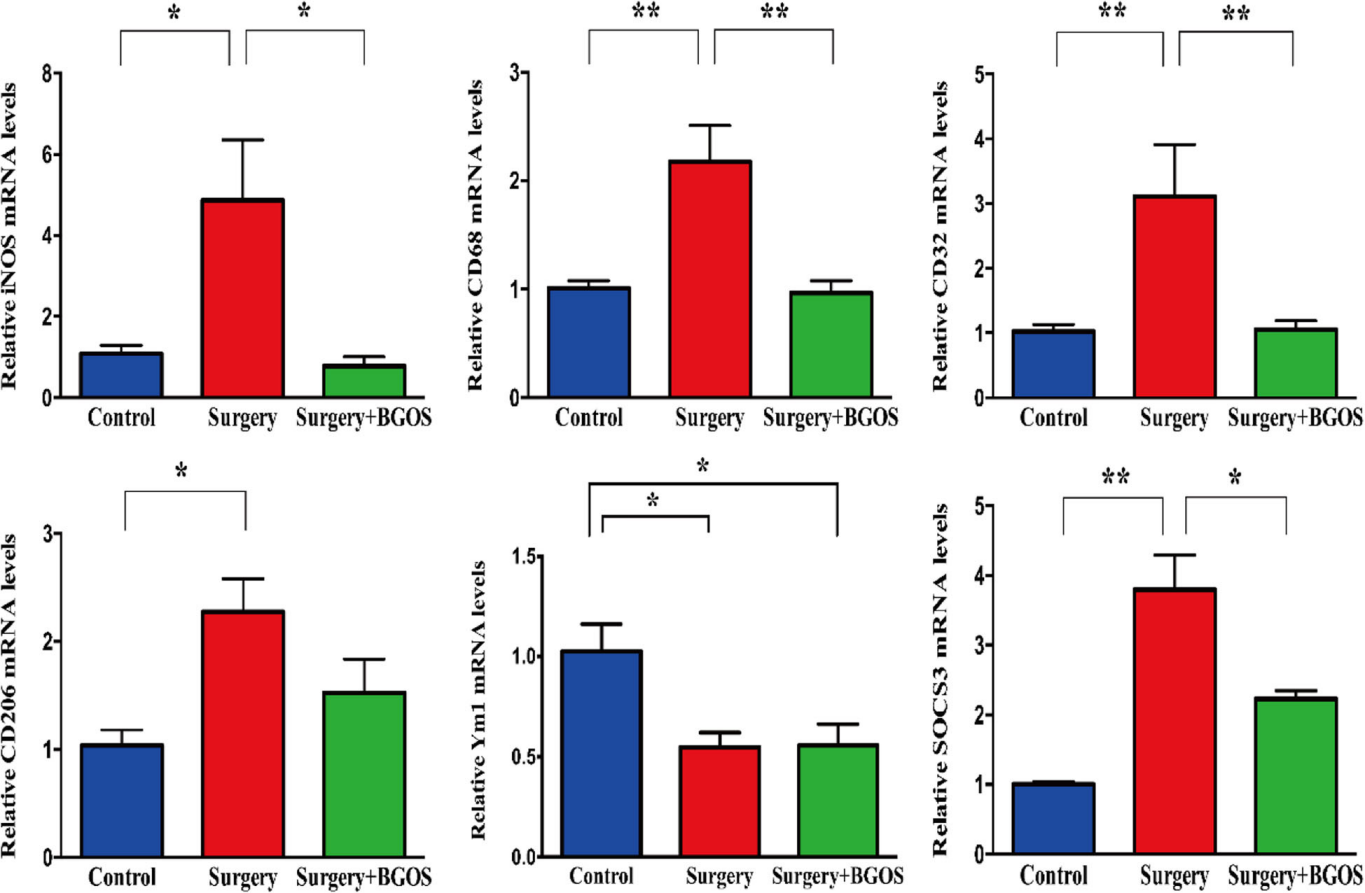
Effects of BGOS on surgery-induced expression of phenotypic genes in the hippocampus. Data are presented as mean ± SEM (*n* = 3–5 per group). *P < .05 and **P < .01 for comparisons shown

Activated microglia are commonly characterized by their signature genes to distinguish between different active phenotypes. In our study, phenotype genes were investigated based on qPCR results. We found that the expression levels of M1-type genes (iNOS, CD68, CD32) in hippocampus were significantly elevated after surgery. Feeding with B-GOS effectively inhibited the increased M1-type gene levels in the hippocampus induced by surgery. However, we observed different trends in expressions of M2-typical genes (CD206, SOCS3, Ym1). Expressions of CD206 and SOCS3 in the surgery group were elevated while Ym1 was reduced. B-GOS downregulated the expression of SOCS3. But, changes in expressions of CD206 and Ym1 induced by surgery were not significantly impacted by B-GOS (Fig. 4).

### Expressions of inflammatory cytokines in hippocampus

**Fig. 5:**
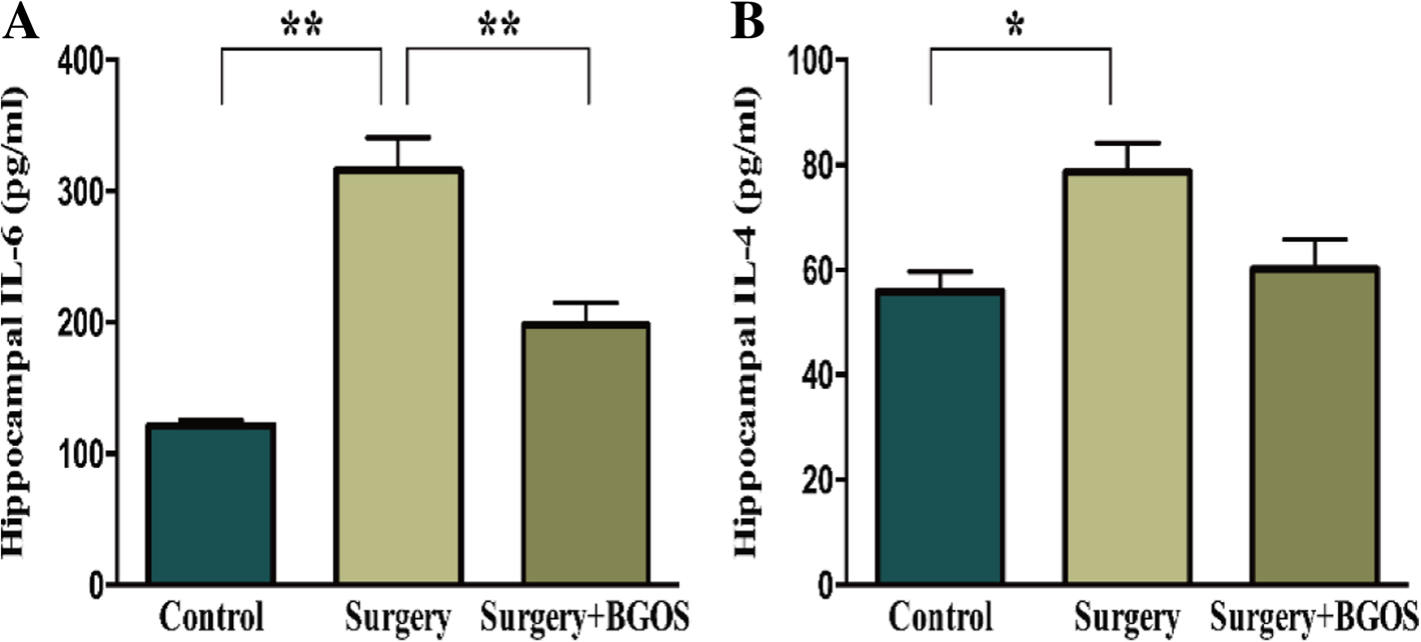
Effects of B-GOS on surgery-induced inflammatory cytokines in the hippocampus. Concentrations of IL-6 (**a**) and IL-4 (**b**) in hippocampus. Data are presented as mean ± SEM (*n* = 3 per group). *P < .05 and **P < .01 for comparisons shown

Protein expressions of IL-6 and IL-4 in rat hippocampus were measured. Hippocampal levels of IL-6 and IL-4 was significantly higher in the surgery group compared with the control group (*P* < .01). B-GOS consumption downregulated the level of IL-6 in hippocampus induced by surgery (*P* < .01). While surgery-induced upregulation of IL-4 was not significantly affected by administration of B-GOS (Fig. 5).

### Microbial community analysis

**Fig. 6:**
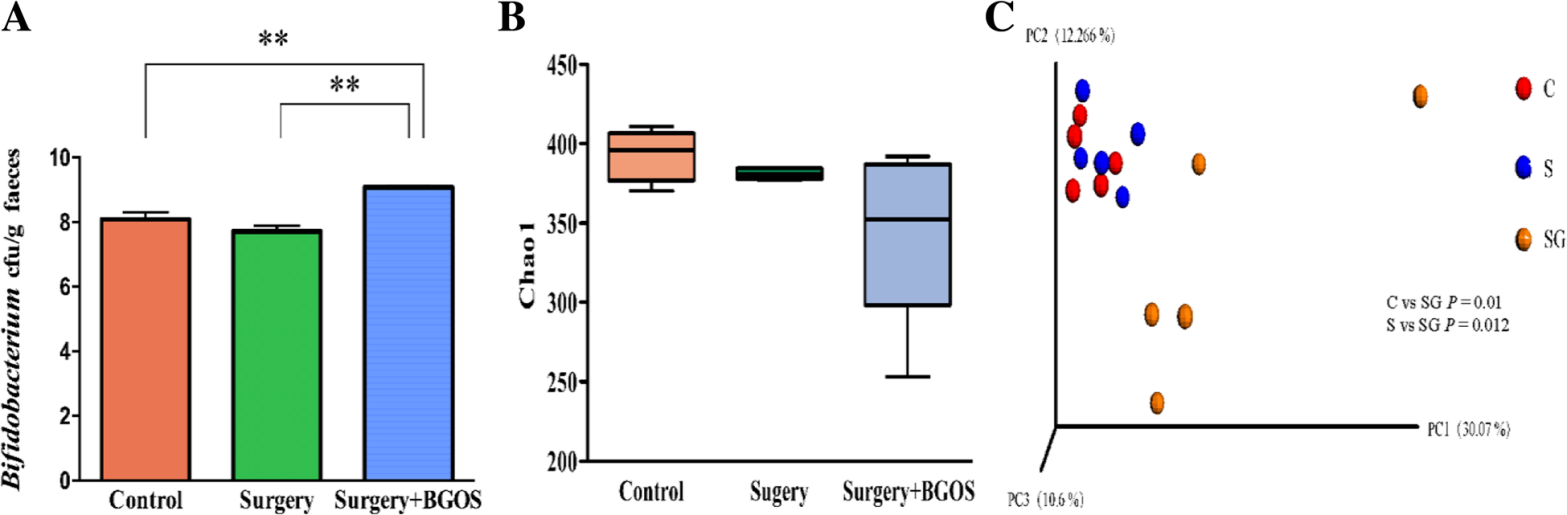
Effects of B-GOS on gut microbiota. (**a**) Absolute quantification of *Bifidobacterium* levels. (**b**) Alpha diversity of the fecal microbiome. (**c**) Principal coordinate analysis. (*n* = 5 per group). **P < .01. C = control group, S = surgery group, SG = surgery + B-GOS group

**Fig. 7:**
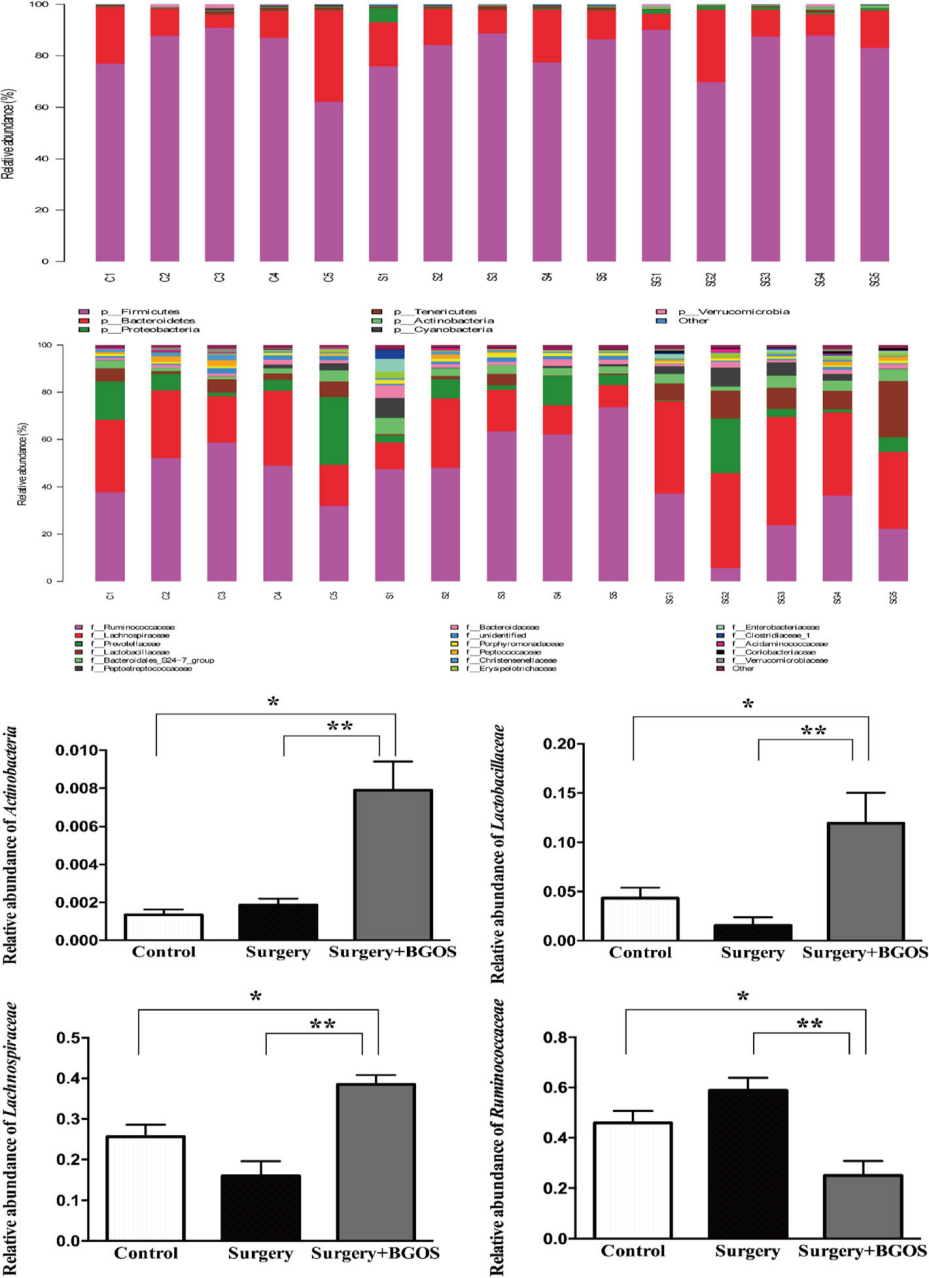
Classification and relative abundance of fecal microorganism at phylum and family levels. (*n* = 5 per group). *P < .05, **P < .01. C = control group, S = surgery group, SG = surgery + B-GOS group

qPCR results showed a higher concentration of *Bifidobacterium* in the B-GOS groups than in animals in the control and surgery groups (*P* < .01) (Fig. 6A). Taxonomy of fecal microbiota was assessed to observe alterations in fecal microbiota using high-throughput analysis of 16S RNA. There was no significant difference between the three groups in the values of Chao1 index (Fig. 6B). Beta diversity was calculated and principal coordinate analysis was performed. Fecal microbiota from B-GOS-fed rats and normal drinking water-fed rats could be significantly separated by principal coordinate analysis (Fig. 6C). Further, we compared fecal microbiota composition between groups at multiple taxonomic levels. At the phylum level, the relative abundance of Actinobacteria was significantly increased in B-GOS-fed rats. At family level, *Lactobacillaceae* and *Lachnospiraceae* were significantly more abundant in the fecal microbiota of rats fed with B-GOS than in those given normal drinking water, while *Ruminococcaceae* was less abundant in the B-GOS group (Fig. 7).

## Discussion

In the present study, we found that: (1) abdominal surgery-induced decline in objective recognition was partially alleviated by administering B-GOS to rats; (2) B-GOS suppressed microglial overactivation and decreased the proportion of M1 phenotypic-microglia induced by surgery; (3) feeding with B-GOS exerted an adequate prebiotic role in promoting proliferation of potentially anti-inflammatory microbes, which may have contributed to regulation of the neuroinflammatory response induced by surgery through the microbiota-brain-axis.

The process of anesthesia and surgery is a strong and acute inflammatory initiator. It triggers a systemic inflammatory response and provokes release of circulatory inflammatory mediators [27]. Overexpression of these inflammatory cytokines disrupts the blood-brain barrier and induces microglial activation and a neuroinflammatory reaction [28, 29]. Exaggerated neuroinflammation is associated with damage of synapses and neurons, and leads to POCD [4]. In this process, activation of microglia plays a major role in neuroinflammation. Increasing evidence indicates that POCD is associated with over-activation of microglia [30]. The hippocampus is the most vulnerable region during the neuroinflammatory response [6]. Consistent with previous studies, an increased expression of Iba-1 and pro-inflammatory cytokines in the hippocampus paralleled by cognitive decline after surgery were observed in the present study.

Main aims of our study were to clarify whether inhibition of surgery-induced neuroinflammation by administering prebiotics is a potential treatment for POCD. Gut microbiota are important for healthy function of the immune and endocrine systems, and can affect various subsets of immune cells. Thus, regulation of the microbiota can impact the host inflammatory response [31]. For the brain, increasing attention is being paid to how the gut microbiota impacts behavior and neuroinflammation. Studies in germ-free mice have shown that the microbiota regulates the blood–brain-barrier and is essential for hippocampal neurogenesis [32]. Erny et al. found that the gut microbiota is requisite for maintaining the steady-state conditions of microglia. In their study, both germ-free and antibiotic-treated mice showed impaired microglial function. Moreover, expressions of some phenotypic genes were changed in the absence of microbiota [33]. Based on the growing findings, strategies are emerging for shifting the composition of gut microbiota via non-dietary or dietary interventions to manipulate neuroinflammation in neuropsychologic disease. The immunoregulatory effect of prebiotics has been proven in previous studies. Savignac et al. revealed that mice fed with prebiotics prevented LPS-induced anxiety and normalized the elevation of IL-1beta level in the brain [21]. Other studies have shown a beneficial role of prebiotic treatment for stress- and beta-amyloid-induced neuroinflammation and cognitive impairment [34, 35]. Therefore, investigations of microbiota­based interventions to resolve surgery-induced neuroinflammation and cognitive dysfunction are warranted. In the present study, we found that the prebiotic B-GOS attenuated surgery-induced cognitive dysfunction and overactivation of microglia.

The presence of multiple activation phenotypes for microglia is an important concept in understanding the role of microglia in inflammation. These different activated states can be characterized and identified by cellular surface receptors or specific gene markers. M1 phenotypic microglia are injurious and detrimental to neurologic outcomes as shown in many models of neurologic diseases, including POCD [8, 36]. In agreement with previous studies, our investigation found that trauma from surgery induced an increased expression of M1-related markers in the hippocampus, and treatment with B-GOS significantly attenuated these increased levels of M1-related markers in the hippocampus following surgery. Production of inflammatory related cytokines was also investigated to identify the M1/M2 phenotypic microglia from the viewpoint of functions. IL-6 has been considered an important pro-inflammatory cytokine in pathogenesis of POCD [37]. Furthermore, IL-6 is thought to be secreted specifically by M1 microglia [38]. Consistent with the profile of M1-related gene markers, the present study found B-GOS feeding could depress the elevation of IL-6 induced by surgery. Therefore, these results suggest that consumption of B-GOS appears to attenuate activation of M1-subpopulations of microglia in surgery-induced neuroinflammatory response.

We also explored the expression of M2-typical genes and cytokines. SOCS3 is a gene marker of M2-immunomodulatory (M2b/c) microglia, which is thought to be a key negative regulator of IL-6 signaling [39]. A previous in vitro study found that pro-inflammatory stimuli induced M1 markers in parallel with the M2-immunomodulatory markers included SOCS3 [40]. Our study also indicated changes in expression of SOCS3 paralleled by the expression of M1-related markers and levels of IL-6. Ym1 and CD206 have been widely investigated M2-typical markers that are involved in tissue remodeling and enhancement of phagocytosis (M2a) [9]. In our study, post-surgical expression of CD206 was elevated while the expression of Ym1 was reduced. However, changes in expression of CD206 and Ym1 induced by surgery were not significantly impacted by B-GOS. IL-4 is a M2a-related microglial cytokine and has been found to play a beneficial role in surgery-induced neuroinflammation and cognitive dysfunction [41]. In the present study, however, surgery-induced increased expression of IL-4 was not affected by administration of B-GOS. Taken together, these findings show that inhibition of M1 activation by B-GOS is mainly involved in the improvement of cognition after surgery while the effect of B-GOS on M2 phenotypic microglia needs more study to clarify the role of prebiotics on microglial phenotypes in POCD as well as on other neuroinflammatory diseases.

To further elucidate the underlying mechanism of B-GOS on POCD and neuroinflammation targeting the brain-gut axis, we collected the feces of rats for microbial community analysis. *Bifidobacterium* is an “immunobiotic” that can beneficially regulate the neuroinflammatory response and behavior in many models of neuroinflammatory-related diseases [42, 43]. The properties of B-GOS have been consistently shown to selectively increase *Bifidobacterium* in both humans and animals [44–46]. This bifidogenic action of B-GOS was enhanced in our study. PCR analysis revealed a significant growth of *Bifidobacterium* after administration of B-GOS. Further, we performed high-throughput analysis of 16S RNA in order to investigate the diversity and bacterial community structure of rats. Alpha diversity was applied to analyze the richness of microbial taxa in a sample. Beta diversity was used to measure the magnitude of differences in community composition among the samples. We did not find significant differences in Chao1 index. This suggests that administration of B-GOS did not affect species richness of the fecal microbiota in rats. However, PCoA results indicated that gut microbiota of B-GOS fed rats were significantly distinct from the other two groups. These results suggest that the effects of prebiotic B-GOS are attributed to overall regulation of intestinal flora rather than to stimulation of a specific type of microorganism. In addition, based on high-throughput analysis, OTU abundance and taxonomic profiles indicated a significantly increased abundance of Actinobacteria, *Lactobacillus,* and *Lachnospiraceae* and a decrease in abundance of *Ruminococcus. Lactobacillus* is a widely studied microbial strain that has beneficial effects on neuroinflammatory-related disease. *Lactobacillus* potently protects memory deficit and neuroinflammation in mouse model of aging and Alzheimer disease [47, 48]. *Lactobacillus* consumption has been found to restore cognition in obese-insulin resistant rats and decrease overactivation of microglia in the hippocampus [17]. Studies have found that *Lachnospiraceae* play an essential role in regulation of gut immune homeostasis. *Lachnospiraceae* depletion was found in the rodent model of inflammation. Furthermore, *Lachnospiraceae* showed negative correlation with place recognition memory in the rat model of high-energy diet-induced cognitive impairment [49]. Burokas et al. found that combined administration of fructooligosaccharides (FOS) and GOS induced an increase in relative abundance of *Lactobacillus* and a decrease in relative abundance of *Ruminococcus*. These prebiotic effects of FOS and GOS could inhibit stress-induced hippocampal inflammation and attenuated stress-related behaviors across domains relevant to anxiety, depression, and cognition [34]. Another investigation also demonstrated that treatment with prebiotic FOS from the medicinal plant *Morinda officinalis* can promote an increase in *Lactobacillus* population and reduce the population of *Ruminococcus* and ameliorate the memory deficiencies and neuroinflammation induced by Aβ through targeting of the microbiota-brain axis [35]. These findings support the notion that the positive effects on the beneficial microbes of prebiotics have a positive role in the neuroinflammatory response and behavior impairments. To our knowledge, the present study is the first to demonstrate that the use of prebiotics to modulate the gut composition of microbiota has beneficial effects on surgery-induced neuroinflammatory response and cognitive dysfunction.

There are several limitations of the present study that must be considered. First, we did not establish a separate animal group exposed to anesthesia alone. Although previous studies have suggested that inhaled anesthetics may induce impairment in learning and memory, other studies have shown that the anesthetic method employed in our study was not associated with any adverse effects on cognition and neuroinflammatory response [27, 50, 51]. Second, we evaluated cognitive function in rats at a single time point rather than a period that includes complete surgical recovery. This test point was selected based on the results of our small-sized pilot experiment. Furthermore, similar results from other studies using a similar surgery model to observe POCD found that cognitive dysfunction was most pronounced within the first week after surgery [6]. Third, we only used the novel objective task as a tool to evaluate the changes of postoperative cognition. This behavior test does not require any external motivation, reward, or punishment and does not generate stressful conditions. In addition, delay of the time interval between the exploration and the recognition phase is more suitable to detect the hippocampus-dependent cognition [52]. However, many other brain regions are involved in the formation and processing of learning and memory, including the cortex and amygdala [53, 54]. Impairment of these brain areas is associated with changes in cognition in models of POCD [7, 50]. Thus, it is necessary to perform a more well-designed protocol and appropriate behavior test to completely evaluate the cognitive impairment and effects of prebiotics in POCD.

## Conclusions

In summary, our data provides laboratory evidence for the beneficial role of the prebiotic B-GOS on surgery-induced neuroinflammation and cognitive dysfunction in early postsurgery through the gut-brain axis. More important, this study supports the potential for a new therapy for POCD in the field of nutritional neuropsychopharmacology.

## Abbreviations

B-GOS: Bimuno®galacto-oligosaccharide
CNS: central nervous system
ELISA: enzyme-linked immunosorbent assay
iNOS: inducible nitric oxide synthase
OTUs: operational taxonomic units
PBS: phosphate buffer solution
PCoA: principal coordinate analysis
POCD: postoperative cognitive dysfunction
qPCR: quantitative polymerase chain reaction
SOCS3: suppressor of cytokine signaling 3
Ym1: chitinase-3 like protein
